# Validation of Self-Management Screening (SeMaS), a tool to facilitate personalised counselling and support of patients with chronic diseases

**DOI:** 10.1186/s12875-015-0381-z

**Published:** 2015-11-11

**Authors:** Nathalie Eikelenboom, Ivo Smeele, Marjan Faber, Annelies Jacobs, Frank Verhulst, Joyca Lacroix, Michel Wensing, Jan van Lieshout

**Affiliations:** Radboud University Medical Centre, Radboud Institute for Health Sciences, IQ healthcare, P.O. Box 9101, 114, 6500, HB Nijmegen, The Netherlands; DOH care group, P.O. Box 704, , 5600, AS Eindhoven, The Netherlands; Doen en blijven doen, Icaruslaan 39, 5631 LH Eindhoven, The Netherlands; Philips Research, High Tech Campus 34, 5656 AE Eindhoven, The Netherlands

**Keywords:** Self-management, Personalisation, Personalised medicine, Chronic care, Primary care, Validation

## Abstract

**Background:**

A rising number of people with chronic conditions is offered interventions to enhance self-management. The responsiveness of individuals to these interventions depends on patient characteristics. We aimed to develop and validate a tool to facilitate personalised counselling and support for self-management in patients with chronic diseases in primary care.

**Methods:**

We drafted a prototype of the tool for Self-Management Screening (SeMaS), comprising 27 questions that were mainly derived from validated questionnaires. To reach high content validity, we performed a literature review and held focus groups with patients and healthcare professionals as input for the tool. The characteristics self-efficacy, locus of control, depression, anxiety, coping, social support, and perceived burden of disease were incorporated into the tool. Three items were added to guide the type of support or intervention, being computer skills, functioning in groups, and willingness to perform self-monitoring. Subsequently, the construct and criterion validity of the tool were investigated in a sample of 204 chronic patients from two primary care practices. Patients filled in the SeMaS and a set of validated questionnaires for evaluation of SeMaS. The Patient Activation Measure (PAM-13), a generic instrument to measure patient health activation, was used to test the convergent construct validity.

**Results:**

Patients had a mean age of 66.8 years and 46.6 % was female. 5.9 % did not experience any barrier to self-management, 28.9 % experienced one minor or major barrier, and 30.4 % two minor or major barriers. Compared to the criterion measures, the positive predictive value of the SeMaS characteristics ranged from 41.5 to 77.8 % and the negative predictive value ranged from 53.3 to 99.4 %. Crohnbach’s alpha for internal consistency ranged from 0.56 to 0.87, except for locus of control (α = 0.02). The regression model with PAM-13 as a dependent variable showed that the SeMaS explained 31.7 % (r^2^ = 0.317) of the variance in the PAM-13 score.

**Conclusions:**

SeMaS is a short validated tool that can signal potential barriers for self-management that need to be addressed in the dialogue with the patient. As such it can be used to facilitate personalised counselling and support to enhance self-management in patients with chronic conditions in primary care.

**Electronic supplementary material:**

The online version of this article (doi:10.1186/s12875-015-0381-z) contains supplementary material, which is available to authorized users.

## Background

The number of people suffering from one or more chronic conditions is rising rapidly. In the Netherlands, the percentage of patients that suffered from at least one chronic disease raised from 12.6 % in 2003 to 15.0 % in 2009 [1]. Several studies have shown positive effects of self-management programs on lifestyle and clinical outcomes, as well as on patients’ motivation, cognition, knowledge, engagement and activation related to self-management behaviours [2–5]. Self-management is defined as ‘the care taken by individuals towards their own health and well-being: it comprises the actions they take to lead a healthy lifestyle; to meet their social, emotional and psychological needs; to care for their long-term condition; and to prevent further illness or accidents’ [6].

However, realising effective self-management in daily life is a major challenge for many people. Substantial variation exists regarding the responsiveness of individuals to self-management interventions [7, 8]. One of the main reasons for the lack of success of self-management interventions is that they often fail to address the individual characteristics of the patient (i.e., personal barriers, needs and situation that play a role in self-management behaviour), resulting in suboptimal reach and impact. The challenge is to find ways to enhance the impact of self-management programs by personalised counselling and support.

The concept of personalisation of treatment to individual characteristics has been introduced in other fields of research, especially human genetics, where treatment is tailored to genomic profiles. This is called personalised medicine [9]. Similarly, in psychology treatment can be adjusted to psychological characteristics of the patient [10–12]. Personalisation has also been applied to web-based interventions and printed material [13, 14]. We applied the concept of personalisation on the application of self-management interventions and hypothesised that self-management counselling and support can be more successful if a personalised approach is used.

Several studies have identified a single determinant of patients’ self-management behaviour, such as self-efficacy, depressive symptoms or coping behaviour [7, 15–17]. Self-efficacy is the patients’ perceived ability to overcome difficulties in behavioural change. This is a predictor for behavioural change, which is needed when confronted with a chronic illness [18, 19]. Co-morbid depression is often seen in patients with chronic diseases [20]. Depression negatively affects patients’ self-care [20]. Finally, coping refers to the way chronically ill patients cope with problems they encounter. An active coping style positively affects well-being and clinical outcomes [21].

It is essential to have a good understanding of the set of individual characteristics that need to be considered in personalised counselling and support of patients’ self-management and how these characteristics can be measured in a valid and feasible way in a primary care setting. There are some instruments available that measure one or more aspects of self-management, often disease specific, such as the Confidence in diabetes self-care scale, or the Nijmegen Clinical Screening Instrument (NCSI) for patients with Chronic Obstructive Pulmonary Disease (COPD) [22, 23]. The NCSI questionnaire measures disease-specific characteristics that determine the health status. The Self-Management Ability Scale (SMAS) focuses on self-management ability of the elderly in relation to well-being [24]. Other instruments have been developed to assess the effect of self-management interventions, such as the Health education impact Questionnaire (heiQ), containing items on health directed behaviour, attitude, self-monitoring and social support [25]. The Partners in Health (PIH) scale and the Patient Activation Measure (PAM-13) both measure the current status of self-management, with items on e.g. knowledge of the condition and skills to monitor symptoms [8, 26].

However, to the best of our knowledge, no generic instrument exists that aims to measure patients’ characteristics that could be a barrier for self-management, in order to provide personalised counselling and support. Moreover, no generic instrument exists that measures these characteristics in a concise way with minimal respondent burden. As with other patient-reported outcome measures, minimising respondent burden is important for the response rate and quality of the collected data [27, 28]. Also, to make the tool practically applicable to facilitate personalised counselling, results should be presented in a way that is easy to interpret and use in day-to-day care [28].

Relying on these insights, we aimed to develop and validate a generic, brief and practically applicable self-management screening questionnaire (SeMaS) to measure possible patient-related barriers to self-management in chronic patients in primary care.

## Methods

A step-wise, mixed methods approach was used to develop and validate the SeMaS tool, as described below. Table 1 provides an overview of the methods used per type of validity. The research ethics committee of Arnhem-Nijmegen reviewed the methods and questionnaires and waived approval.

**Table 1:** Overview of methods used for validation of SeMaS

Type of validation	Method
Content validity	Literature review
	Focus group interviews
Face validity	Focus group interviews
	Stakeholders group
Criterion validity	Calculation of PPV, NPV and correlations of SeMaS using the validated questionnaires as ‘golden standard’
(Convergent) Construct validity (hypothesis testing)	Correlation of SeMaS with PAM-13
Reliability: internal consistency	Crohnbach’s alpha

## Setting

The study was carried out in two primary care group practices in the south of the Netherlands. These practices are a member of ‘De Ondernemende Huisarts’ (DOH), an innovative primary care group comprising 15 primary care group practices, and serving approximately 110.000 patients. The care group provides integrated healthcare for several chronic diseases, and has defined self-management as a priority in their policy. The professionals of this care group are all trained in the ‘actual practice and maintenance’ approach, consisting of a behaviour change model (“series of steps”) and “individual related factors” to enhance self-management [29]. The “individual related factors” include the constructs locus of control, self-efficacy, anxiety, depression, stress, coping style, styles of attribution, pain and somatisation. These factors can be barriers for behavioural change.

## Content validity and face validity

The content and face validity of SeMaS were based on a literature review, focus group interviews with patients and healthcare professionals, and pilot testing.

### Literature review

Many publications report on one or two individual characteristics that influence self-management behaviour. To create an overview of possible important patient-related characteristics, we performed a review in 2011 based on a systematic literature search in the PubMed and psycINFO databases, and screening of articles from the years 2000 to 2011 on title and abstract. Due to time constraints, we were not able to screen all full texts. Therefore, of the 133 articles we screened all full texts that described trials, controlled or comparative research, and the most recent non-comparative, or qualitative research. In total, 68 articles were screened, and from 42 full-text articles, all the characteristics that were named in the articles were listed, as well as instruments used for measurement. This review yielded a list of 43 items, many of which overlapped. Examples were sex, age, perceived burden of disease, and psychosocial factors such as self-efficacy, social support and coping. Details of the literature review are provided in Additional file 1.

### Focus groups

Focus group interviews were held to document what patients and professionals considered important for effective self-management based on their experience. A purposeful sample of patients with chronic conditions (the same patients participated in 3 focus groups; *n* = 10, *n* = 6, *n* = 5) and primary care professionals (including general practitioners, psychologist, dietician, physiotherapist, nurse; 2 focus groups; *n* = 4, *n* = 5, one GP participated in both groups) participated in the focus groups. The items from the literature review were used as input in these interviews. The focus group interviews were audio recorded, and field notes were made for identification of the important characteristics. A report was made of each focus group, which was sent to the participants as preparation for the next session. Details of the focus group interviews are described in Additional file 1. The result of the focus group interviews was a preliminary list of characteristics that should be incorporated in the tool, according to the patients and professionals.

### Stakeholder group

The development of the tool was guided by a group of stakeholders, consisting of two general practitioners, a psychologist, a nurse, three researchers, and two healthcare innovation experts of two health insurance companies. After completing the focus group interviews, the stakeholder group made the final selection of characteristics to be incorporated in the tool, as described below. Also, the stakeholder group monitored whether the study was conducted as planned.

### Prototype questionnaire

Combining the results of the literature study with those from the focus groups, the stakeholder group selected the characteristics that should be incorporated in the tool, as shown in Table 2. Considering the application in a primary care setting, we wished to develop a short, user-friendly tool, which could be used across chronic conditions and types of patients. Characteristics were selected if a) the characteristic was named in the focus group interviews, b) we found scientific evidence in the literature study that this characteristic influenced self-management, and c) a validated tool was available to measure the characteristic. A few exceptions were made. Age (date of birth) and education, known predictors of health literacy, were already incorporated in the questionnaire to gather demographic data. Therefore, health literacy, the ability to read and understand things people commonly encounter in the healthcare setting, was not incorporated in the tool, as to reduce the length of the questionnaire [30]. Sense of self-esteem and confidence in self-care were two characteristics similar to self-efficacy, and were therefore not incorporated in the tool. Two additional constructs were incorporated in the tool. Locus of control, the belief of a person that the attainment of a certain outcome is within (internal locus) or outside (external locus) their own control, was mentioned in the focus group interviews and the stakeholders group [19]. Anxiety was also mentioned in the stakeholders group, as a characteristic that has impact on self-management. Therefore, anxiety and locus of control were incorporated in the tool.

**Table 2:** Selection of characteristics for the SeMaS questionnaire

Patient characteristics identified in literature review	Mentioned in focus group interviews with professionals and/or patients	Scientific evidence for impact on self-management	Validated measure available	Included in SeMaS	Number of items in SeMaS
Educational level	Yes	1, 2	Yes	Yes	1
Sex	Yes	2	Yes	Yes	1
Age	Yes	2	Yes	Yes	1
Perceived burden of disease	Yes	2	Yes (inverse)	Yes	1
Self-efficacy	Yes	1, 2	Yes	Yes	2
Social support	Yes	1, 2	Yes	Yes	1 (6 subitems)
Depression/depressive symptoms	Yes	1, 2	Yes	Yes	3
Health literacy	Yes	2	Yes	No	-
Coping	Yes	2	Yes	Yes	9
Sense of self esteem	Yes	2	No	No	-
Confidence in self care	Yes	2	No	No	-
Locus of control	Yes	-	Yes	Yes	2
Anxiety	No (added by stakeholders)	-	Yes	Yes	4

Level of scientific evidence: 1 = comparative research; 2 = non-comparative, or qualitative research. Only characteristics that were named in the focus groups or stakeholder group and that were included in the questionnaire are shown here. See Additional file 1 for the entire list of characteristics found in the literature

Next, we developed a prototype of the questionnaire to test in practice. For the selected characteristics, we used items from validated questionnaires, which we identified in the literature review, further targeted searches on the Internet, or consultation of experts in the stakeholders group. The items were selected, considering the psychometric features of the questionnaire if available. Otherwise, items were selected based on face validity, as described in the Measures section. Specifically, the research team discussed whether the selected items would suffice to cover all aspects of the construct being measured. For uniformity in the newly developed SeMaS questionnaire, the wording of some items in SeMaS was slightly altered. For example: all items were formulated in I-form instead of the you-form. We also slightly adjusted the response scales to make it as uniform as possible, and thus easier for the patient to fill out the questionnaire. For example, the response scale of self-efficacy was reduced from a 7 point to a 4 point Likert scale, and still ranging from completely false to completely true. Also, one option was added to the response scale of coping, ranging from never to very often/continuously, to correspond with the anxiety and depression subscales. However, when scoring these items, the extra option for coping (‘often’) received the same score as the option ‘very often/continuously’. As a result, all questions of SeMaS had a 4 or 5 point Likert scale, except perceived burden of disease. This item was scored on a visual analogue scale from 0 to 10. The original questionnaires are provided in Additional file 2. To enhance the content validity, the stakeholders group assessed the prototype, leading to some minor adaptations. Three items were added to guide the type of support or intervention, being computer skills, functioning in groups, and willingness to perform self-monitoring.

### Pilot testing

**Fig. 1:**
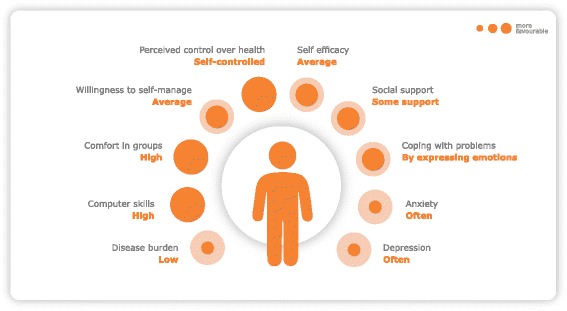
Example of the SeMaS graphic profile as a representation of the scores on SeMaS

Before starting the observational study, we tested the prototype SeMaS questionnaire on a small scale. The readability and feasibility in general practice were pre-tested in the third focus group with patients. After minor textual adjustments, the questionnaire was tested in two group practices of the DOH care group. Twenty-four patients with chronic conditions participated while waiting for their consultation with the practice nurse. Patients received information about the study, and informed consent was obtained. Patients were asked to fill in the questionnaire. Results were displayed in a graphic profile; a visual representation of the scores on SeMaS, as displayed in Fig. 1. With this profile, the healthcare professional is able to see the results at a glance. This profile was discussed with the patient during the consult. After the consultation, interviews were held with the patients to identify problems with the readability and feasibility of the questionnaire, resulting in some small textual adjustments to the questionnaire. Also, the effect of discussing the profile during the consult was evaluated during the interview.

### Interpretability

We wanted to provide the results of SeMaS in a graphic profile that can be easily used and interpreted by the healthcare professional in day-to-day care. For each characteristic, a score was calculated by adding up the scores of the individual items. These scores were categorised and presented in a graphic profile in terms of barriers for self-management: no, minor, or major barriers. For anxiety and depression, the categorisation was in line with the original questionnaire, where the categories no, minor and major elevation were used based on the sum scores. For locus of control and coping, the categorisation in the type of locus (internal or external) or coping style (problem solving, expressing emotions or looking for distraction) was used. No categorisation was available for perceived burden of disease, self-efficacy, and social support. Therefore, categorisation was based on face validity. For perceived burden of disease, a low burden (0–2) was classified as a possible minor barrier for self-management, as the motivation for behavioural change could be low. On the other hand, a high burden of disease (8–10) could hinder self-management due to e.g. functional impairments, and thus also classified as minor barrier. Scores 3 to 7 were classified as no barrier.

The responses for the two items on self-efficacy were scored from 0 to 3, thus having a maximum sum score of 6. The scores 4 to 6 were classified as having high self-efficacy and thus no barrier, 2 to 3 as a minor barrier, and 0 to 1 as major barrier.

For social support, the responses ‘no’, ‘completely false’ and ‘somewhat false’ were scored as 0; ‘somewhat true’ as 1, and ‘completely true’ as 2. Total score for this category was 12. The scores 3 to 12 were categorised as having no barrier (thus having 2–3 persons in the patients’ network who can provide support), 2 was categorised as having a minor barrier, and 1 to 0 as having a major barrier.

Although developed separately, the SeMaS questionnaire and the ‘actual practice and maintenance approach’ overlapped substantially in patient characteristics that were included for their importance for self-management, such as self-efficacy, social support, anxiety, depression, coping and locus of control [29]. Therefore, a manual was developed for the practice nurses to guide the interpretation and use of this information, based on the ‘actual practice and maintenance approach’ [29]. Both the categorisation and manual were developed in collaboration with the developer (author FV) of this approach.

## Construct and criterion validity

The construct and criterion validity of the SeMaS tool were tested in an observational study with a sample of patients from two general practices.

### Study population

The patient sample comprised patients visiting two group practices from the care group. Patients with a chronic disease (diabetes mellitus, COPD, asthma, and/or (high risk for) cardiovascular disease) who were treated for this condition in primary care by a practice nurse, and were over 18 years of age were eligible to participate in the study. The chronic conditions were coded according to the International Classification of Primary Care (ICPC) in the medical health records.

To test the practicality of the instrument, practice nurses were asked to discuss the results of SeMaS with the patients in planned consultations. To simulate the normal procedure in the primary care practice, patients were selected from the agenda of the practice nurse. As many patients as possible were selected per day in a five month period, while not selecting on other patient characteristics, besides the inclusion criteria. 243 patients responded to the invitation to participate in the study.

Patients were invited to complete the questionnaire and return it in a prepaid envelope to the research institute, together with the signed informed consent form. The questionnaire consisted of the SeMaS, and a set of validated questionnaires to evaluate SeMaS, as described in the ‘Measures’ section.

## Measures

### SeMaS

The SeMaS questionnaire that was tested contained 27 items in total, measuring the psychological constructs self-efficacy (2 items), coping (6 items), depression (3 items), anxiety (4 items) and locus of control (2 items) [18, 19]. The construct of social support included 5 items [31]. Also, the perceived burden of disease was assessed (1 item). Three other items that guide the type of support concerned computer skills, functioning in groups and willingness to perform self-monitoring (3 items). The questionnaire was made available in the Dutch language.

### Criterion validity

To determine the criterion validity of the SeMaS, we assessed whether the scores on the SeMaS were an adequate reflection of the scores on the original validated instruments. For this purpose, patients were asked to fill in these original instruments. The original instruments and the items used in SeMaS are described below. The original instruments were used in their original form.

#### Self-efficacy

In the SeMaS questionnaire, two out of four items from the perceived competence scale were used to determine the level of self-efficacy, based on face validity [19, 32, 33]. To improve uniformity in the SeMaS questionnaire, we adjusted the response categories of the original questionnaire from a 7-point to a 4-point Likert scale, ranging from completely false to completely true . Items were translated from English to Dutch by the research team. For assessing the criterion validity of the characteristic self-efficacy, two questions from the Dutch version of the Patient Activation Measure (PAM-13) were used in the analysis, as these items were very similar, and were already assessed for construct validity [8, 34]. The PAM-13 measures knowledge, skills and self-efficacy for self-management [8].

#### Coping

For coping, we used the Dutch version of the short Utrecht Coping List (UCL-k) [18, 35]. This questionnaire consists of 14 items, with a 4-point response scale ranging from never to very often/continuously. Respondents indicate how often they cope with problems in a certain way. The questionnaire distinguishes three coping styles, being problem solving (P), expressing emotions (E) and looking for distraction (D). Based on correlations between the items from available research data from 183 carers for patients with dementia, we used two items per coping style in the SeMaS questionnaire, thus six items in total. The wording of the items was slightly altered to the I-form, and the response categories adjusted to the 5-point scale of the anxiety and depression subscales.

#### Anxiety and depression

For anxiety and depression, we used these subscales of the validated Dutch version of the 4-dimensional symptom questionnaire (4DSQ). The 4DSQ is a self-report questionnaire of 50 items that measures non-specific general distress, depression, anxiety and somatisation, with a 5-point response scale ranging from never to very often/continuously [36]. Using available data from 2127 patients in general practice [36], we computed correlations between the items, and removed items that had a correlation >0.7 with another item. For anxiety, we used 4 from the original 12 items. For depression, we used 3 from the original 6 items. The wording of the questions was slightly changed to the I-form. Response categories were not altered.

#### Locus of control

We used the Dutch version of the Multidimensional Health locus of control scale (MHLCS) for locus of control [19, 37]. The MHLCS consists of 18 questions with a 6-point Likert-scale, ranging from completely disagree to completely agree. The MHLCS identifies three orientation scales, being: physician orientation, chance orientation, and internal orientation. Sum scores are produced per scale. The scale with the highest score is the most prevalent one. In the SeMaS questionnaire we dichotomised the locus of control sum score into an internal orientation versus an external orientation (physician and chance), consistent with the “actual practice and maintenance” approach [29]. We used two MHLCS items in SeMaS that most clearly represented the internal and external orientation. The items were chosen on face validity, as no data was available to us. The response categories were reduced to a 4-point Likert-scale for uniformity in the SeMaS.

#### Social support

For social support, the Short Scale of Social Support (SSSS) was used. This questionnaire consists of 5 items and measures actual support in case of need [38]. One item on the support from neighbours was added, as this was relevant for the Dutch context. The response categories were adjusted to match the subscales of self-efficacy and locus of control for consistency from helpful (not at all-completely) to completely disagree/agree (both 4-point Likert scale). Items were translated from English to Dutch by the research team.

#### Perceived burden of disease

For perceived burden of disease, the Dutch version of the EQ-5D questionnaire was used, containing five dimensions of quality of life, and one item on the perceived health status [39]. The item on the perceived health status was used in the SeMaS, and inversely formulated to measure the perceived burden of disease. The item was scored on a visual analogue scale from 0 to 10 (0 to 100 in EQ-5D), as this is a concise way of measuring subjective characteristics.

### Convergent construct validity

Hibbard *et al.* developed a short questionnaire to measure the level of patient activation, the PAM-13 [8]. This questionnaire assesses patient knowledge, skills, and self-efficacy for self-management [8]. The PAM-13 is one of the few generic measures for the level of self-management. We obtained permission of Insignia to use the PAM-13 in this study.

We used the validated Dutch version of PAM-13 to determine the convergent construct validity, hypothesising that the better the score on the SeMaS (i.e. having fewer barriers to self-management), the higher the score on PAM-13 [34]. We obtained permission of Insignia to use the PAM-13 in this study.

## Data-analysis

Descriptive analysis was used to inspect the distributions of scores and numbers of missing values on items in the SeMaS questionnaire. When computing sum scores for each construct, cases with missing values were excluded, except for social support. In the SeMaS and the Short Scale of Social support the missing values were interpreted as ‘not applicable’.

The internal consistency was determined for each construct in SeMaS using Crohnbach’s alpha. Values of 0.6–0.7 are considered acceptable; 0.7 or higher is considered as good [40].

To determine the criterion validity, the positive (PPV) and negative predictive value (NPV) of the characteristics in SeMaS were determined regarding the scores on the (more comprehensive) original questionnaires. The positive predictive value (PPV) indicates the percentage of rightly detected barriers by SeMaS, compared to the original questionnaire. The PPV is calculated by dividing the number of patients with a barrier for self-management detected by SeMaS and the original questionnaire, by the total number of patients with a barrier detected by SeMaS. The negative predictive value (NPV) indicates the percentage of rightly indicated absence of barriers by SeMaS, compared to the original questionnaire. The NPV is calculated by dividing the number of patients with no barrier detected by SeMaS and the original questionnaire, by the total number of patients with no barrier detected by SeMaS. We considered the PPV and NPV as relevant measures for application of the SeMaS tool in everyday practice [41, 42]. Also, correlations of the sum scores of each construct in SeMaS with the sum scores on the original questionnaire were computed to investigate the relationship between both measures. Values of 0.3 to 0.7 indicate a moderate linear relationship; 0.7 or higher indicate a strong linear relationship. For validation purposes, a correlation of 0.7 is recommended [43]. We checked whether the sum scores were normally distributed. In case of a normal distribution, we used Pearson’s *r*, otherwise the non-parametric Spearman’s rho.

PAM-13 was used to determine the construct validity of the SeMaS based on convergent validity. In a regression analysis, categorical variables should consist of two categories. Therefore, in the regression analysis, we dichotomised the scores on SeMaS into having no barrier versus having a (minor or major) barrier. Using univariate ANOVA, all constructs were tested on whether the PAM scores differed between the categories. Subsequently, using multivariate linear regression models, the relations between the relevant SeMaS characteristics and PAM scores (0–100) were investigated. We performed all analyses using SPSS software (version 20, IBM Corp.).

We performed the aforementioned analyses as primary analyses. Based on the findings in the primary analyses, the tool was adjusted to its final version as a last step in the validation process. After the final adjustments, we performed the secondary analyses. The final instrument is added in Additional file 3, as well as the calculation of the scores. The results of the primary analyses are shown in Additional file 4. Also, the final adaptations are described in this additional file. Here, we present the results of the secondary analyses, as these represent the test characteristics of the instrument in its actual form.

## Results

Of the 243 eligible patients, 204 returned the questionnaire (response rate: 84 %). Of the respondents, 53.4 % was male, with an average age of 66.7 ± 9.1 years, and 46.6 % was female, with an average age of 66.9 ± 9.6 years (Table 3). Most of the participants had multiple ICPC codes for (risk of) chronic conditions. The ICPC codes for (risk of) cardiovascular disease was present for 92.6 and 91.7 % of the male and female participants, respectively. Of the male participants, 16.2 % had a diagnosis of diabetes mellitus, while 35.4 % of the female participants had this diagnosis. The ICPC codes for asthma/COPD were found in 12.0 % of the male participants, and 19.8 % of the female participants. No data was available on the non-responders, as these patients did not give their consent for the study.

**Table 3:** Description of the study population (numbers (%))

	Male	Female
Number of respondents	109 (53.4 %)	95 (46.6 %)
Age in years (mean ± SD)	66.7 ± 9.1	66.9 ± 9.6
Education		
-No education	0 (0 %)	3 (3.2 %)
-Lower education	18 (16.5 %)	31 (32.7 %)
-Middle education	43 (39.4 %)	27 (28.4 %)
-Higher education	42 (38.6 %)	20 (21.0 %)
-Other	1 (0.9 %)	6 (6.3 %)
-Missing	5 (4.6 %)	8 (8.4 %)
Chronic illness		
-Diabetes	49 (16.2 %)	34 (35.4 %)
-CVRM	100 (92.6 %)	88 (91.7 %)
-Asthma/COPD	13 (12.0 %)	19 (19.8 %)
-Other	3 (2.8 %)	6 (6.3 %)
-Missing	2 (1.9 %)	0 (0 %)

The number of missing values per characteristic in SeMaS ranged from 3 (self-efficacy) to 64 (coping). For social support, 101 cases had missing values on the individual items. One case was excluded, which had no valid responses on the items for social support. Cases were excluded from the analysis if they had missing data for the construct being analysed.

**Fig. 2:**
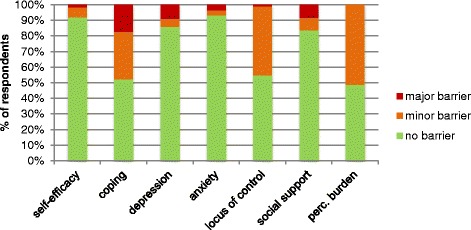
Scores of the study population on the psychological characteristics and social support of SeMaS. The category ‘minor barrier’ of coping includes patients that had multiple coping styles (21.7 %). Perc. burden: perceived burden of disease

In Fig. 2, the distribution of the scores on SeMaS on the psychosocial characteristics and perceived burden of disease is displayed. On separate characteristics, 1.0 to 17.4 % of the patients showed major barriers, and 3.0 to 51.2 % showed minor barriers, according to the SeMaS scores. Only 5.9 % of the patients showed no barrier on any of the characteristics. 28.9 % had one minor or major barrier. This is shown in Table 4.

**Table 4:** Number of respondents with minor or major barriers for self-management

Number of barriers	Number of respondents	Percentage of respondents
0	12	5.9
1	59	28.9
2	62	30.4
3	48	23.5
4	12	5.9
5	8	3.9
6	2	1.0
7	1	0.5
Total	204	100

## Criterion validity

In Table 5, the PPV, NPV and correlation of SeMaS with the sum scores on the original questionnaires are displayed. The PPV and NPV are based on the categories on each characteristic. The PPV ranged from 41.5 to 77.8 %, and the NPV from 53.3 to 99.4 %. Crohnbach’s alpha (α) ranged from 0.56 to 0.86, except for locus of control (α = 0.02). For anxiety (α =0.56) and coping (subtype: looking for distraction; α =0.56), Crohnbach’s alpha was just below the acceptable range. Crohnbach’s alpha (α) was within the acceptable range (0.6–0.7) for social support (α =0.63), and good (>0.7) for self-efficacy (α =0.86), coping (subtypes: problem solving (α =0.70), emotional (α =0.73)), and depression (α =0.87). Correlations of SeMaS sum scores with the original questionnaires ranged from 0.418 to 0.805. Self-efficacy, (0.418, *p* < 0.01), anxiety (0.653, *p* < 0.01), locus of control (0.472, *p* < 0.01), social support (0.626, *p* < 0.01), and perceived burden of disease (0.554, *p* < 0.01) showed a moderate correlation with the original questionnaires. Coping (0.746; 0.800; 0.783, *p* < 0.01) and depression (0.805, *p* < 0.01) showed a strong correlation with the original questionnaires. Spearman’s rho was computed for self-efficacy, depression, anxiety, social support and perceived burden of disease, since the sum scores of these characteristics were not normally distributed.

**Table 5:** Description of the psychometric characteristics of the final SeMaS

Characteristic	n	PPV	NPV	Crohnbach’s alpha (α) for internal consistency	Correlation of sum score on SeMaS with sum score on original questionnaire
Self-efficacy	182	57.1	79.8	0.86	0.418**^a^
Coping	171	41.5	94.4	0.70	0.746** (P)
				0.73	0.800** (E)
				0.56	0.783** (D)
Depression	192	67.9	99.4	0.87	0.805** ^a^
Anxiety	182	77.8	91.3	0.56	0.653** ^a^
Locus of control	147	67.6	53.3	0.02	0.472**
Social support	191	N.A.	N.A.	0.63	0.626**^a^
Perceived burden of disease	163	N.A.	N.A.	N.A.	0.554**^a^

*PPV* percentage of patients with a barrier on SeMaS that has a barrier according to the original questionnaire

*NPV* percentage of patients with no barrier on SeMaS that has no barrier according to the original questionnaire

*N. A.* not applicable

Coping styles: (P) problem solving; (E) emotional; (D) distraction

^a^ correlation: spearman’s rho; sum scores were not normally distributed

* *p* < 0.05; ** *p* < 0.01

## Construct validity

All psychosocial characteristics and perceived burden of disease from the SeMaS showed significant different PAM-scores between the SeMaS categories (no versus minor/major barrier), except coping. These characteristics were used in the regression analysis. With the PAM scale as dependent variable, the forced entry regression showed that these psychosocial characteristics together explained 31.7 % (r^2^ = 0.317) of the variance in the PAM score. The β of the separate characteristics varied from 0.04 for depression (*p* = 0.990) to 16.43 for self-efficacy (*p* <0.001), as displayed in Table 6. Social support (*p* = 0.032) and self-efficacy (*p* <0.001) contributed significantly to the model. The β values for these characteristics indicated that patients without a barrier for self-efficacy scored 16.43 points higher on the PAM than patients with a barrier. Similarly, patients without a barrier for social support scored 6.64 points higher on the PAM.

**Table 6:** Results of the regression model with PAM-13 as the dependent variable

Parameter	β	SE β	Standardised β	*p*
Intercept	34.50			0.000
Locus of control	2.99	2.18	0.10	0.173
Anxiety	0.84	4.97	0.10	0.866
Depression	0.04	3.46	<0.01	0.990
Social support	6.64	3.06	0.16	0.032
Self-efficacy	16.43	2.82	0.46	<0.001
Perceived burden of disease	3.51	2.11	0.12	0.098

## Discussion

This manuscript describes the validation of SeMaS using a mixed methods approach. We aimed at developing a short screening questionnaire that is applicable in day-to-day care by practice nurses in a primary care setting. The instrument is now ready for testing of its impact in every day practice.

The analyses for criterion validity showed reasonable to good results, with values for NPV and PPV that are comparable to or better than other diagnostic tests in primary care [44–46]. Most patients showed barriers for coping and locus of control. The majority of the participants did not experience any anxiety or self-efficacy barriers. The PPV and NPV are dependent on the prevalence of the barriers. A low prevalence causes a low PPV, even when the sensitivity and specificity of the test are good. This could be the case for anxiety, as only 4.9 % of the patients had a barrier detected by SeMaS, and the PPV was on the low side. Thus, SeMaS could be sufficiently able to detect barriers on anxiety, while this is not reflected in the PPV value.

Furthermore, the values of Crohnbach’s alpha for internal consistency are in the acceptable range for most of the characteristics. The value of Crohnbach’s alpha depends on the length and heterogeneity of the construct being measured [47]. This could explain the lower values for the characteristics locus of control, social support and anxiety. These constructs are heterogeneous, e.g. for anxiety several types of anxiety complaints are measured: irrational fears, anticipation, anxiety and avoidance behaviour [36].

For locus of control, both the Crohnbach’s alpha and correlations are low. This could be due to the concise way of measuring this characteristic in the SeMaS, as we decreased the number of items from 18 in the original questionnaire to two items in the SeMaS. Literature on this characteristic showed that the scientific evidence of the impact of locus of control on self-management is inconclusive [15, 48]. Therefore, further validation and research on this characteristic is recommended.

The SeMaS is developed with the explicit aim to contain only a limited number of questions to enable its feasibility in daily practice. Depending on the cut-off points of the categories, more false positive or more false negative cases will be detected. We defined the cut-off points realising that the instrument opens up the opportunity to find barriers otherwise at all undetected. The SeMaS is intended as a tool to signal potential barriers for self-management that need to be addressed in the conversation with the patient, rather than as a diagnostic tool.

Depression and anxiety are important possible barriers for self-management [49], and often undiagnosed by the general practitioner [50–52]. SeMaS helps in detecting these problems. If indicated, additional diagnostic instruments should be used for anxiety and depression.

To test whether the tool was practically applicable, practice nurses were asked to discuss the results of SeMaS with the patients during the planned consults in the observational study. In evaluative interviews, the practice nurses indicated the tool as practical and useful in practice, although they needed time to learn to work with the tool (findings available upon request).

The SeMaS scores explained part of the variance in the PAM-13 scores. The main part of this explained variance could be attributed to the shared characteristic of both instruments, being self-efficacy. As PAM-13 further measures knowledge and skills (related to management of the disease, and not related to possible interventions), which are not incorporated in the SeMaS, it is understandable that not all variance in the PAM scores can be explained by the SeMaS. Nevertheless we chose PAM-13 as a reference, since it is one of the few generic measures for the level of self-management at this time.

As discussed in the introduction, there are a few other instruments that show some overlap with the SeMaS. These instruments assess disease-specific characteristics (NCSI), or focus on the current level of self-management (PIH, PAM-13) [8, 26, 53]. The difference between these instruments and SeMaS is that SeMaS is a generic instrument that assesses person-related characteristics that could be a barrier for self-management of a chronic condition. By assessing these characteristics and presenting the results in a graphic profile, SeMaS can be used to provide personalised counselling and support.

## Strengths and limitations of this study

SeMaS was systematically developed, using a range of methods. Based on the input from the focus group interviews and the stakeholder group much effort was put in the uniformity of the questionnaire regarding wording of the items and response categories. In the first analyses the rephrasing of various items showed to be a factor with negative influence on the PPV and the NPV. For this reason we decided to adjust the wordings more in line with the original questionnaires, as described in Additional file 4. A further strength of this study was that the response of the selected sample was high (>80 %), decreasing the risk of attrition bias. Also, the minimal number of respondents (150) for a validation study was well achieved. Another strength of this study could be that the participating primary care practices are part of an innovative care group, which has formulated a policy agenda on self-management. This may have influenced the development of the instrument in a positive way, as the participating patients and professionals may have been more experienced in what characteristics could hinder self-management. Furthermore, the fact that patients and health care professionals were involved in the development of this instrument increases the possibility of the instrument being applicable in practice.

A limitation of this study is that the literature study comprised a systematic search, and screening of all abstracts for inclusion, but review of a subset of the full text articles. Since many characteristics were mentioned in multiple articles, and the characteristics that are important for self-management were also discussed in the focus group interviews, we felt we captured the most important characteristics that had to be incorporated in the tool.

Another limitation of this study could be that the participating primary care practices are part of an innovative care group, with a policy agenda on self-management. Possibly, the issues raised by the patients and professionals in the focus groups differ from issues in other care groups. This may have implications for use in practice, as other care groups less experienced with stimulating self-management may not recognise the incorporated characteristics as important, or may have less possibilities in providing personalised support.

## Conclusions

The SeMaS questionnaire has been developed and validated in this study. The SeMaS is now ready for testing in practice as a generic, brief, practically applicable tool to measure possible patient-related barriers to self-management in chronic patients in primary care.

The results of this study show that it is possible to create profiles of patients regarding their self-management competence. Next, we will investigate the applicability and the impact of the use of these profiles in counselling on self-management in primary care [54]. Using the profiles to provide personalised self-management support may positively influence the effectiveness of the support and self-management interventions. This next study will also provide possibilities for further validation of the SeMaS questionnaire.

## Additional files

Additional file 1:
**Description of the literature study and focus groups.** Describes the methods and results of the literature study and focus groups, on which the final selection of characteristics that should be incorporated in the SeMaS questionnaire was based. (PDF 352 kb) Additional file 2:
**Original questionnaires and examples of adaptations of the original items in SeMaS.** Provides the original questionnaires, and examples of the original items, and the adapted items in SeMaS. (PDF 398 kb) Additional file 3:
**SeMaS questionnaire and scoring categories.** Provides the translated version of the final SeMaS questionnaire. It also provides the scoring categories that were used to determine the presence of no, a minor or a major barrier for each characteristic in SeMaS. (PDF 177 kb) Additional file 4:
**Results of primary analysis of SeMaS.** Provides the results of the primary analysis of SeMaS with the criterion measures and PAM-13. (PDF 110 kb)

## References

[1] Tacken MA, Opstelten W, Vossen I, Smeele IJ, Calsbeek H, Jacobs JE (2011). Increased multimorbidity in patients in general practice in the period 2003–2009. Ned Tijdschr Geneeskd.

[2] de Silva D, Evidence: Helping people help themselves. A review of the evidence considering whether it is worthwhile to support self-management. Health Foundation (Great Britain), 2011, ISBN 978–1–906461–26–3.

[3] Effing T, Monninkhof EM, van der Valk PD, van der Palen J, van Herwaarden CL, Partidge MR (2007). Self-management education for patients with chronic obstructive pulmonary disease. Cochrane Database Syst Rev.

[4] Orrow G, Kinmonth AL, Sanderson S, Sutton S (2012). Effectiveness of physical activity promotion based in primary care: systematic review and meta-analysis of randomised controlled trials. BMJ.

[5] Lu Z, Cao S, Chai Y, Liang Y, Bachmann M, Suhrcke M (2012). Effectiveness of interventions for hypertension care in the community--a meta-analysis of controlled studies in China. BMC Health Serv Res.

[6] Department of Health (2005). Self Care - A Real Choice: Self Care Support - A Practical Option.

[7] Bischoff EW, Hamd DH, Sedeno M, Benedetti A, Schermer TR, Bernard S (2011). Effects of written action plan adherence on COPD exacerbation recovery. Thorax.

[8] Hibbard JH, Mahoney ER, Stockard J, Tusler M (2005). Development and testing of a short form of the patient activation measure. Health Serv Res.

[9] Ziegler A, Koch A, Krockenberger K, Grosshennig A (2012). Personalized medicine using DNA biomarkers: a review. Hum Genet.

[10] Cuijpers P, Reynolds CF, Donker T, Li J, Andersson G, Beekman A (2012). Personalized treatment of adult depression: medication, psychotherapy, or both? A systematic review. Depress Anxiety.

[11] Siskind D, Harris M, Pirkis J, Whiteford H (2012). Personalised support delivered by support workers for people with severe and persistent mental illness: a systematic review of patient outcomes. Epidemiol Psychiatr Sci.

[12] Evers AW, Rovers MM, Kremer JA, Veltman JA, Schalken JA, Bloem BR (2012). An integrated framework of personalized medicine: from individual genomes to participatory health care. Croat Med J.

[13] Noar SM, Benac CN, Harris MS (2007). Does tailoring matter? Meta-analytic review of tailored print health behavior change interventions. Psychol Bull.

[14] Lustria ML, Cortese J, Noar SM, Glueckauf RL (2009). Computer-tailored health interventions delivered over the Web: review and analysis of key components. Patient Educ Couns.

[15] Ahola AJ, Groop PH (2013). Barriers to self-management of diabetes. Diabet Med.

[16] Dirik G, Karanci AN (2010). Psychological distress in rheumatoid arthritis patients: an evaluation within the conservation of resources theory. Psychol Health.

[17] Murray J, Fenton G, Honey S, Bara AC, Hill KM, House A (2013). A qualitative synthesis of factors influencing maintenance of lifestyle behaviour change in individuals with high cardiovascular risk. BMC Cardiovasc Disord.

[18] Kratz AL, Molton IR, Jensen MP, Ehde DM, Nielson WR (2011). Further evaluation of the Motivational Model of Pain Self-Management: coping with chronic pain in multiple sclerosis. Ann Behav Med.

[19] AbuSabha R, Achterberg C (1997). Review of self-efficacy and locus of control for nutrition- and health-related behavior. J Am Diet Assoc.

[20] Thomas J, Jones G, Scarinci I, Brantley P (2003). A descriptive and comparative study of the prevalence of depressive and anxiety disorders in low-income adults with type 2 diabetes and other chronic illnesses. Diabetes Care.

[21] Scharloo M, Kaptein AA, Weinman J, Hazes JM, Willems LN, Bergman W (1998). Illness perceptions, coping and functioning in patients with rheumatoid arthritis, chronic obstructive pulmonary disease and psoriasis. J Psychosom Res.

[22] Van Der Ven NC, Weinger K, Yi J, Pouwer F, Ader H, Van Der Ploeg HM (2003). The confidence in diabetes self-care scale: psychometric properties of a new measure of diabetes-specific self-efficacy in Dutch and US patients with type 1 diabetes. Diabetes Care.

[23] Vercoulen JH (2012). A simple method to enable patient-tailored treatment and to motivate the patient to change behaviour. Chron Respir Dis.

[24] Schuurmans H, Steverink N, Frieswijk N, Buunk BP, Slaets JP, Lindenberg S (2005). How to measure self-management abilities in older people by self-report. The development of the SMAS-30. Qual Life Res Int J Qual Life Asp Treat Care Rehab.

[25] Osborne RH, Elsworth GR, Whitfield K (2007). The Health Education Impact Questionnaire (heiQ): an outcomes and evaluation measure for patient education and self-management interventions for people with chronic conditions. Patient Educ Couns.

[26] Petkov J, Harvey P, Battersby M (2010). The internal consistency and construct validity of the partners in health scale: validation of a patient rated chronic condition self-management measure. Qual Life Res Int J Qual Life Asp Treat Care Rehab.

[27] Turner RR, Quittner AL, Parasuraman BM, Kallich JD, Cleeland CS, Mayo FDAP-ROCMG (2007). Patient-reported outcomes: instrument development and selection issues. Value Health.

[28] Aaronson N, Alonso J, Burnam A, Lohr KN, Patrick DL, Perrin E (2002). Assessing health status and quality-of-life instruments: attributes and review criteria. Qual Life Res Int J Qual Life Asp Treat Care Rehab.

[29] van der Burgt M, Verhulst F (2009). Doen en blijven doen.

[30] Baker DW, Williams MV, Parker RM, Gazmararian JA, Nurss J (1999). Development of a brief test to measure functional health literacy. Patient Educ Couns.

[31] Strom JL, Egede LE (2012). The impact of social support on outcomes in adult patients with type 2 diabetes: a systematic review. Curr Diab Rep.

[32] Williams GC, McGregor HA, Zeldman A, Freedman ZR, Deci EL (2004). Testing a self-determination theory process model for promoting glycemic control through diabetes self-management. Health Psychol.

[33] Williams GC, Lynch M, Glasgow RE (2007). Computer-assisted intervention improves patient-centered diabetes care by increasing autonomy support. Health Psychol.

[34] Rademakers J, Nijman J, van der Hoek L, Heijmans M, Rijken M (2012). Measuring patient activation in The Netherlands: translation and validation of the American short form Patient Activation Measure (PAM13). BMC Public Health.

[35] Schreurs PJG, van de Willige G, Brosschot JF, Tellegen B, Graus GMH (1993). De Utrechtse Copinglijst: UCL. Omgaan met problemen en gebeurtenissen.

[36] Terluin B, van Marwijk HW, Ader HJ, de Vet HC, Penninx BW, Hermens ML (2006). The Four-Dimensional Symptom Questionnaire (4DSQ): a validation study of a multidimensional self-report questionnaire to assess distress, depression, anxiety and somatization. BMC Psychiatr.

[37] Wallston KA, Wallston BS, DeVellis R (1978). Development of the Multidimensional Health Locus of Control (MHLC) Scales. Health Educ Monogr.

[38] Funch DP, Marshall JR, Gebhardt GP (1986). Assessment of a short scale to measure social support. Soc Sci Med.

[39] Salen BA, Spangfort EV, Nygren AL, Nordemar R (1994). The Disability Rating Index: an instrument for the assessment of disability in clinical settings. J Clin Epidemiol.

[40] Kline P (2000). The handbook of psychological testing.

[41] Knottnerus JA, Leffers P (1992). The influence of referral patterns on the characteristics of diagnostic tests. J Clin Epidemiol.

[42] Schneider A, Gindner L, Tilemann L, Schermer T, Dinant GJ, Meyer FJ (2009). Diagnostic accuracy of spirometry in primary care. BMC Pulm Med.

[43] Terwee CB, Bot SD, de Boer MR, van der Windt DA, Knol DL, Dekker J (2007). Quality criteria were proposed for measurement properties of health status questionnaires. J Clin Epidemiol.

[44] Cadogan A, McNair P, Laslett M, Hing W (2013). Shoulder pain in primary care: diagnostic accuracy of clinical examination tests for non-traumatic acromioclavicular joint pain. BMC Musculoskelet Disord.

[45] Carballido J, Fourcade R, Pagliarulo A, Brenes F, Boye A, Sessa A (2011). Can benign prostatic hyperplasia be identified in the primary care setting using only simple tests? Results of the Diagnosis IMprovement in PrimAry Care Trial. Int J Clin Pract.

[46] Damian AM, Jacobson SA, Hentz JG, Belden CM, Shill HA, Sabbagh MN (2011). The Montreal Cognitive Assessment and the mini-mental state examination as screening instruments for cognitive impairment: item analyses and threshold scores. Dement Geriatr Cogn Disord.

[47] Schmitt N (1996). Uses and abuses of coefficient alpha. Psychol Assess.

[48] Wiebe JS, Christensen AJ (1996). Patient adherence in chronic illness: Personality and coping in context. J Pers.

[49] Gazmararian JA, Ziemer DC, Barnes C (2009). Perception of barriers to self-care management among diabetic patients. Diabetes Educator.

[50] Janssen EH, van de Ven PM, Terluin B, Verhaak PF, van Marwijk HW, Smolders M (2012). Recognition of anxiety disorders by family physicians after rigorous medical record case extraction: results of the Netherlands Study of Depression and Anxiety. Gen Hosp Psychiatry.

[51] Smolders M, Laurant M, van Wamel A, Grol R, Wensing M (2008). What determines the management of anxiety disorders and its improvement?. J Eval Clin Pract.

[52] Hermanns N, Caputo S, Dzida G, Khunti K, Meneghini LF, Snoek F (2013). Screening, evaluation and management of depression in people with diabetes in primary care. Primary care diabetes.

[53] Peters JB, Daudey L, Heijdra YF, Molema J, Dekhuijzen PN, Vercoulen JH (2009). Development of a battery of instruments for detailed measurement of health status in patients with COPD in routine care: the Nijmegen Clinical Screening Instrument. Qual Life Res Int J Qual Life Asp Treat Care Rehab.

[54] Eikelenboom N, van Lieshout J, Wensing M, Smeele I, Jacobs AE (2013). Implementation of personalized self-management support using the self-management screening questionnaire SeMaS; a study protocol for a cluster randomized trial. Trials.

