# Supplementary material for: Validation of Self-Management Screening (SeMaS), a tool to facilitate personalised counselling and support of patients with chronic diseases
Source: BMC Fam Pract. 2015 Nov 11;16:165. doi: 10.1186/s12875-015-0381-z (PMC4641335; doi:10.1186/s12875-015-0381-z)
Supplement: Additional file 1: — Description of the literature study and focus groups. Describes the methods and results of the literature study and focus groups, on which the final selection of characteristics that should be incorporated in the SeMaS questionnaire was based. (PDF 352 kb) [file 12875_2015_381_MOESM1_ESM.pdf]

Additional file 1. Description of the literature study and focus groups

Literature study

A comprehensive literature search was conducted in the databases of PubMed and psychINFO over the years 2000-2011, using the search terms: Chronic illness(es) or Chronic disease(s), COPD, asthma, diabetes mellitus, cardiovascular disease(s), self-management or self-care, typology or category or model or taxonomy, psychological adaptation or coping or adaptive behaviour or health attitude or health behaviour. We retrieved 256 articles.

Two independent authors performed the screening of the articles on title and abstract for inclusion. Inclusion criteria were: 1) the study objects are adults or adolescents; 2) the study contains a typology or classification of patients, or contains a patient model, or contains patient characteristics in relation to self management or self care; 3) the study objects are patients with at least one chronic disease. Exclusion criteria were: 1) the study objects are not adults or adolescents; 2) the study provides a typology of people in general; 3) the study provides a typology or classification of the disease and not of the patient. In the case of disagreement, the authors discussed the abstract. If no consensus was reached, the article was included for full text screening. In total, 133 articles were included for full text screening. Second, the full texts were screened on characteristics that influenced the ability to perform self-management behaviour either positive or negative, and the level of evidence based on the study design. Due to time constraints, we were not able to screen all full texts. Therefore, we screened all full texts that described trials, controlled or comparative research (level 0 or 1), and the most recent non-comparative, or qualitative research (2). In total, 68 articles were screened.

The full text screening yielded a list of 43 characteristics, many of which overlapped. The characteristics are shown in Table 1.

Table 1. Selection of characteristics for the SeMaS questionnaire from the literature

Characteristic derived from literature	Mentioned in focus group interviews with professionals and/or patients	Scientific evidence for impact on self-management	Validated measure available	Final selection
Ethnicity	Yes	1, 2		
Educational level	Yes	1, 2	Yes	Yes
Gender	Yes	2	Yes	Yes
Age	Yes	2	Yes	Yes
Occupation		2	Yes	
Income		2	Yes	
Duration of the disease		2	Yes	
BMI		2	Yes	
Blood pressure		2	Yes	
Early onset of complications		2	Yes	
Number of medications		2	Yes	
Comorbidity		2	Yes	
Perceived burden of disease	Yes	2	Yes	Yes
Self efficacy	Yes	1, 2	Yes	Yes
Social support	Yes	1, 2	Yes	Yes
Depression/depressive symptoms	Yes	1, 2	Yes	Yes
Diabetes distress		1, 2	Yes	
Cognitive change processes		1		
Change processes of behaviour		1		
Decisional balance		1		
competence	Yes	2		
Experienced control		2		
attitude	Yes	2		
Activity status		2		
Health literacy	Yes	2	Yes	
coping	Yes	2	Yes	Yes
Skills	Yes	2		Yes
Trust		2		
Self monitoring behaviour		2		
Experience		2		
Perceived importance		2		
Emotions due to disease		2		
Behaviour related to physical activity		2		
Sense of self esteem	Yes	2		
Identity		2		
Knowledge		2		

Overprotective partner		2		
Beliefs about heart failure		2		
Primary and secondary cognitive assessment of the diagnosis		2		
Personal history and social context		2		
Subjective norms		2		
Tasks for adjustment to the disease		2		
Confidence in self care	Yes	2		

Level of scientific evidence: 1=comparative research; 2=non-comparative, or qualitative research.

Focus groups interviews

Patients

For the focus group interviews, a group of patients was recruited from several general practices of the care group. The same patients participated in the different focus groups. In the first focus group interview (n=10 patients), we presented existing models of self-management, including the “actual practice and maintenance” approach consisting of the behaviour change model “series of steps” and “person related factors”, and the general model of self-management developed by the Dutch national action program self-management [1, 2]. Patients were asked what they thought is important in self-management of a chronic illness. Patients mentioned the presence of social support, motivation, perceived burden of disease, knowledge as characteristics that could positively influence the level of self-management. Also, skills were important to perform self-management. Pain and anxiety could negatively influence the level of self-management according to the panel.

In the second focus group interview (n=6 patients), characteristics from the literature study were discussed with the patients. From the list of characteristics, patients named educational level, social context, and perceived burden of disease as important. To test existing questionnaires on relevance and difficulty, patients were asked to fill in validated questionnaires on different characteristics: general self-efficacy scale [3], appraisal of self-care agency [4], Oslo 3-item social support scale [5], and the anxiety and depression

subscales of the four dimensional symptom questionnaire (4DSQ)[6]. The main feedback of the patients was that several questionnaires were about situations in general. According to the patients, the answers on the questions would be different if the questions were about health goals or lifestyle.

To test the readability of the SeMaS questionnaire, the patients were asked to fill in a prototype in a third focus group interview (n=5 patients). The questionnaire was evaluated with the patients, resulting in minor textual adjustments.

Healthcare professionals

In the first focus group interview with professionals (n=4 professionals: general practitioners, physiotherapist, dietician) we also presented the existing models, and the characteristics from the literature. Professionals were asked to prioritise the characteristics on level of importance for self-management of a chronic illness, and to provide arguments for this prioritisation. Professionals concluded that the following characteristics should be in the instrument: perceived self efficacy, social support, psychological well being/depression, health literacy, perceived burden of disease, level of education, and skills. Additionally, locus of control was discussed as an important characteristic, closely related to self efficacy, coping, and psychological well being.

In the second focus group interview (n=5 professionals: general practitioners, nurse, psychologist), one general practitioner from the first focus group participated. The results from the first focus group were presented, and opinions on the results were assessed. Subsequently, available questionnaires were reviewed and judged on their validity, comprehensiveness and usefulness in practice. Professionals concluded that the following characteristics should be in the instrument: social support, motivation/self efficacy, coping, competence (and skills), depression, and attitude. Also, we discussed possible missing characteristics from the list. The professionals named locus of control, anxiety, and health literacy as important characteristics for self management. Computer skills were thought to be important for the type of intervention to stimulate self-management.

The final selection of the characteristics for the SeMaS instrument is described in the main manuscript.

Literature

1. Burgt. MVd, Verhulst. F: **Doen en blijven doen**, 4 edn: Springer; 2009.
2. Zelfmanagement LA: **Generiek Model Zelfmanagement**. In.: CBO; 2011: 2.
3. Bosscher RJ, Smit JH: **Confirmatory factor analysis of the General Self-Efficacy Scale**. *Behaviour research and therapy* 1998, **36**(3):339-343.
4. Evers GC, Isenberg MA, Philipsen H, Senten M, Brouns G: **Validity testing of the Dutch translation of the appraisal of the self-care agency A.S.A.-scale**. *International journal of nursing studies* 1993, **30**(4):331-342.
5. Dalgard OS: **Community health profile as tool for psychiatric prevention**. In: *Promotion of mental health.* Edited by Trent DR, Reed CA, vol. 5: Avebury; 1996: 429.
6. Terluin B, van Marwijk HW, Ader HJ, de Vet HC, Penninx BW, Hermens ML, van Boeijen CA, van Balkom AJ, van der Klink JJ, Stalman WA: **The Four-Dimensional Symptom Questionnaire (4DSQ): a validation study of a multidimensional self-report questionnaire to assess distress, depression, anxiety and somatization**. *BMC psychiatry* 2006, **6**:34.
