# Supplementary material for: Validation of Self-Management Screening (SeMaS), a tool to facilitate personalised counselling and support of patients with chronic diseases
Source: BMC Fam Pract. 2015 Nov 11;16:165. doi: 10.1186/s12875-015-0381-z (PMC4641335; doi:10.1186/s12875-015-0381-z)
Supplement: Additional file 2: — Original questionnaires and examples of adaptations of the original items in SeMaS. Provides the original questionnaires, and examples of the original items, and the adapted items in SeMaS. (PDF 398 kb) [file 12875_2015_381_MOESM2_ESM.pdf]

Additional file 2 – Original questionnaires and examples of adaptations of the original items in SeMaS

In this Additional file, we present the original questionnaires, as well as some examples of the original items and adapted items in SeMaS.

Multidimensional Health Locus of Control scale [1]
Response scale: 7-point Likert Scale, from Completely disagree to Completely agree.

Internal orientation
1. If I get sick, it's my own behavior which determines how soon I get well again.
6. I am in control of my health.
8. When I get sick I am to blame.
12. The main thing which affects my health is what I myself do.
13. If I take care of myself, I can avoid illness.
17. If I take the right actions, I can stay healthy.

Powerful others
3. Having regular contact with my physician is the best way for me to avoid illness.
5. Whenever I don't feel well, I should consult a medically trained professional.
7. My family has a lot to do with my becoming sick or staying healthy.
10. Health professionals control my health.
14. When I recover from an illness, it's usually because other people (for example, doctors, nurses, family, friends) have been taking good care of me.
18. Regarding my health, I can only do what my doctor tells me to do.

Chance
2. No matter what I do, if I am going to get sick, I will get sick.
4. It seems that my health is greatly influenced to me by accident.
9. Luck plays a big part in determining how soon I will recover from an illness.

Perceived competence scale [2]
Response scale: 7-point, from 'not at all true' to 'very true'.
The items of this scale are formulated according to the issue at hand, in this example diabetes.
1.I feel confident in my ability to manage my diabetes.
2.I am capable of handling my diabetes now.
3.I am able to do my own routine diabetic care now.
4.I feel able to meet the challenge of controlling my diabetes.

Short Scale of Social Support [3]
Response scale: 4-point Likert Scale, from 'not at all helpful' to very helpful.

Introduction:
When a person is sick, other people sometimes
help and sometimes hinder them, whatever their intentions
are. I am going to mention some people and I would like
you to tell me how much help they have been to you.
Spouse
Children
Other relatives

Friends
Coworkers

Coping [4]
Response scale: 4-point Likert scale, from ‘never’ to ‘very often’.

1. Uw ergernis laten blijken
2. Laten zien dat u kwaad bent op degene die verantwoordelijk is voor het probleem
3. Direct ingrijpen als er moeilijkheden zijn
4. Uw zorgen tijdelijk verdrijven door er even uit te gaan
5. Afleiding zoeken
6. Een probleem van alle kanten bekijken
7. Verschillende mogelijkheden bedenken om een probleem op te lossen
8. Doelgericht te werk gaan om een probleem op te lossen
9. De zaken eerst op een rij zetten
10. Aan andere dingen denken die niet met het probleem te maken hebben
11. Op één of andere manier proberen u prettiger te voelen
12. Uw gevoelens tonen
13. Troost en begrip zoeken
14. Laten merken dat u ergens mee zit

4-Dimensional Screening Questionnaire [5]
Response scale: 5 point Likert scale, from ‘never’ to ‘very often’.

Anxiety
During the past week, did you suffer from:
1. sudden shock for no reason?
2. indefinable feelings of fear?
3. trembling when with other people?
4. anxiety or panic attacks?
During the past week, did you feel:
5. frightened?
During the past week, did you:
6. have any fear of going out of the house alone?
During the past week:
7. were you afraid of anything when there was really no need for you to be afraid? (for instance animals, heights, small rooms)
8. were you afraid to travel on busses, trains or trams?
9. were you afraid of becoming embarrassed when with other people?
10. did you ever feel as if you were being threatened by unknown danger?
11. did you have to avoid certain places because they frightened you?
12. did you have to repeat some actions a number of times before you could do something else?

Depression
During the past week, did you feel:
1. that everything is meaningless?
2. that life is not worth while?
3. that you would be better off if you were dead?
4. that you can't enjoy anything anymore?
5. that there is no escape from your situation?

During the past week:
6. did you ever think "If only I was dead"?

Perceived burden of disease [6]
The EQ-5D questionnaire is copyright protected. Please visit the website www.euroqol.org for more information.

Examples of adaptations of the original items in SeMaS
Here, we provide some examples of the items from the original questionnaires, and the adapted items used in SeMaS to illustrate the magnitude of the adaptations. The numbers at the response options represent the score for that option.

Example 1: coping
Original items:

	**No**	**Sometimes**	**Often**	**Very often/ Always**
Think of other things that have nothing to do with the problem	☐0	☐1	☐2	☐2
Show your feelings	☐0	☐1	☐2	☐2

Items in SeMaS:

	**No**	**Sometimes**	**Often**	**Frequentl y**	**Always**
I think of other things that have nothing to do with the problem	☐0	☐1	☐2	☐2	☐2
I show my feelings (e.g. anger, sadness)	☐0	☐1	☐2	☐2	☐2

Example 2: Locus of control
Original items:

Locus of control	**Disagree strongly**	**Disagre e**	**Somewh at disagree**	**Some what agree**	**agre e**	**Agree stron gly**
The main thing which affects my health is what I myself do.	☐6	☐5	☐4	☐3	☐2	☐1
Regarding my health, I can only do what my doctor tells me to do.	☐6	☐5	☐4	☐3	☐2	☐1

Items in SeMaS:

Locus of control	**Disagree strongly**	**Disagree**	**Agree**	**Agree strongly**
The main thing which affects my health is what I myself do.	☐0	☐1	☐2	☐3
Regarding my health, I can only do what my doctor tells me to do.	☐3	☐2	☐1	☐0

References

1. Wallston KA, Wallston BS, DeVellis R: **Development of the Multidimensional Health Locus of Control (MHLC) Scales**. *Health education monographs* 1978, **6**(2):160-170.
2. Williams GC, McGregor HA, Zeldman A, Freedman ZR, Deci EL: **Testing a self-determination theory process model for promoting glycemic control through diabetes self-management**. *Health psychology : official journal of the Division of Health Psychology, American Psychological Association* 2004, **23**(1):58-66.
3. Funch DP, Marshall JR, Gebhardt GP: **Assessment of a short scale to measure social support**. *Social science & medicine* 1986, **23**(3):337-344.
4. Schreurs PJG, G.; vdW, J.F.; B, B.; T, G.H.M. G: **De Utrechtse Copinglijst: UCL. Omgaan met problemen en gebeurtenissen**. Lisse: Swets & Zeitlinger; 1993.
5. Terluin B, van Marwijk HW, Ader HJ, de Vet HC, Penninx BW, Hermens ML, van Boeijen CA, van Balkom AJ, van der Klink JJ, Stalman WA: **The Four-Dimensional Symptom Questionnaire (4DSQ): a validation study of a multidimensional self-report questionnaire to assess distress, depression, anxiety and somatization**. *BMC psychiatry* 2006, **6**:34.
6. Salen BA, Spangfort EV, Nygren AL, Nordemar R: **The Disability Rating Index: an instrument for the assessment of disability in clinical settings**. *Journal of clinical epidemiology* 1994, **47**(12):1423-1435.
