# Supplementary material for: Validation of Self-Management Screening (SeMaS), a tool to facilitate personalised counselling and support of patients with chronic diseases
Source: BMC Fam Pract. 2015 Nov 11;16:165. doi: 10.1186/s12875-015-0381-z (PMC4641335; doi:10.1186/s12875-015-0381-z)
Supplement: Additional file 3: — SeMaS questionnaire and scoring categories. Provides the translated version of the final SeMaS questionnaire. It also provides the scoring categories that were used to determine the presence of no, a minor or a major barrier for each characteristic in SeMaS. (PDF 177 kb) [file 12875_2015_381_MOESM3_ESM.pdf]

Additional file 3. SeMaS questionnaire and scoring categories.

A. SeMaS questionnaire

SeMaS: Self Management Screening

Fill-in date ………-……….-………………….. (dd-mm-yyyy)

Date of birth ………-……….-………………….. (dd-mm-yyyy)

Gender
- ☐ Male
- ☐ Female

1. What is your highest completed education?

- ☐ No education
- ☐ Elementary school
- ☐ Preparatory education
- ☐ Junior general secondary education
- ☐ Intermediate vocational education
- ☐ Senior general secondary education or Pre-University education
- ☐ Higher vocational education or university
- ☐ Other: ______________________________

2. Please answer the following question

How much burden do you experience usually from your disease(s)?

|—————————————————————————————|

0 1 2 3 4 5 6 7 8 9 10

No burden at all Unbearably high burden

	Skills	**Disagree strongly**	**Disagree**	**Agree**	**Agree strongly**
3.	I have good computer skills (e.g. looking up on google, e-mail)	☐	☐	☐	☐
4.	I function well in groups (e.g. colleagues, other patients etc.)	☐	☐	☐	☐
5.	I am prepared to perform self care (e.g. measure blood pressure, weighing)	☐	☐	☐	☐

© IQ Healthcare

	Locus of control	Disagree strongly	Disagree	Agree	Agree strongly
6.	The main thing which affects my health is what I myself do.	☐	☐	☐	☐
7.	Regarding my health, I can only do what my doctor tells me to do.	☐	☐	☐	☐

	Self-efficacy	Disagree strongly	Disagree	Agree	Agree strongly
8.	I think I am able to live in a healthy way (e.g. eating healthy, excercise adequately, no smoking)	☐	☐	☐	☐
9.	If I put effort into it, I will be able to live in a healthy way (e.g. eating healthy, excercise adequately, no smoking)	☐	☐	☐	☐

Social support

10.			These persons are helpful when I have health problems			
I have the following persons in my network:			Completely false	Somewhat false	Somewhat true	Completely true
Partner	☐No	Yes ->	☐	☐	☐	☐
Children	☐No	Yes ->	☐	☐	☐	☐
Other relatives	☐No	Yes ->	☐	☐	☐	☐
Neighbours	☐No	Yes ->	☐	☐	☐	☐
Friends	☐No	Yes ->	☐	☐	☐	☐
Colleagues	☐No	Yes ->	☐	☐	☐	☐

Coping
When people experience problems, they usually react more often in one way than the other. Please indicate after each sentence **how often you in general react in the way that is described** when you experience problems.

		No	Sometimes	Often	Frequently	Always
11.	I think of other things that have nothing to do with the problem	☐	☐	☐	☐	☐
12.	I show my feelings (e.g. anger, sadness)	☐	☐	☐	☐	☐
13.	I try to feel better in some way	☐	☐	☐	☐	☐
14.	I think of several possibilities to solve the problem	☐	☐	☐	☐	☐
15.	I look for distraction	☐	☐	☐	☐	☐
16.	I search for comfort and understanding	☐	☐	☐	☐	☐
17.	I work purposeful to solve the problem	☐	☐	☐	☐	☐
18.	I let other notice that I have a problem with something	☐	☐	☐	☐	☐
19.	I intervene directly when problems occur	☐	☐	☐	☐	☐

Anxiety and depression

In the next questions, it is about complaints and symptoms that you experienced **in the past week (the last 7 days, including today).** Complaints you had before, but not during the past week, do not count. Please indicate per complaint how often you experienced this in the last week by checking the corresponding box.

		No	Sometimes	Often	Frequently	Always
**During the past week, I had**						
20.	anxiety or panic attacks	☐	☐	☐	☐	☐
**During the past week, I was:**						
21.	afraid of leaving the house alone	☐	☐	☐	☐	☐
22.	afraid of something I shouldn’t be afraid of (e.g. heights, animals, small spaces)	☐	☐	☐	☐	☐
23.	scared of being embarrassed in company of other people	☐	☐	☐	☐	☐

	**During the past week, I had the feeling**					
24.	that I could not have fun anymore	☐	☐	☐	☐	☐
25.	that everything was pointless	☐	☐	☐	☐	☐
26.	that life wasn’t worth living	☐	☐	☐	☐	☐

B. Scoring and categories of SeMaS.

The items of SeMaS are scored and subsequently sum scores are computed for each characteristic. Overall, the items are scored from zero to four. The perceived burden of disease is scored on a visual analogue scale from zero to 10. For coping, depression and anxiety, all responses from ‘often’ to ‘always’ are scored as 2. Item 7 is inversely scored.
The cut off values for the categories were based on the original questionnaires, or on face validity if no categorisation was available. Table 1 provides the cut off values based on the sum scores per characteristic on the SeMaS questionnaire. For coping, the sum score per coping style is determined. The style with the highest score is the most prevalent one. All characteristics have three categories, except perceived burden of disease, which has two categories.

Table 1. Cut off values for categories of SeMaS

Characteristic	Major barrier	Minor barrier	No barrier
Perceived burden of disease	-	0-2/8-10	3-7
Locus of control	0-1	2-3	4-6
Self-efficacy	0-1	2-3	4-6
Social support	0-1	2	3-12
Coping	D	E	P
Anxiety	4-8	3	0-2
Depression	3-6	2	0-1

Coping styles: (P) problem solving; (E) emotional; (D) distraction.

Coping styles: (P) problem solving; (E) emotional; (D) distraction.
