# Supplementary material for: Validation of Self-Management Screening (SeMaS), a tool to facilitate personalised counselling and support of patients with chronic diseases
Source: BMC Fam Pract. 2015 Nov 11;16:165. doi: 10.1186/s12875-015-0381-z (PMC4641335; doi:10.1186/s12875-015-0381-z)
Supplement: Additional file 4: — Results of primary analysis of SeMaS. Provides the results of the primary analysis of SeMaS with the criterion measures and PAM-13. (PDF 110 kb) [file 12875_2015_381_MOESM4_ESM.pdf]

Additional file 4. Results of primary analysis of SeMaS

In this Additional file, we show the results of the analyses on the SeMaS data prior to the minor adjustments we made. Also, we describe which adjustments we made. First, we computed the positive (PPV) and negative (NPV) predictive values per characteristic, comparing SeMaS with the original questionnaires, as shown in Table 1. To determine the internal consistency, we computed Crohnbach's alpha.

Table 1. Description of the psychometric characteristics of the SeMaS from the primary analysis

Characteristic	PPV	NPV	Crohnbach's alpha (α) for internal consistency	Correlation with sum score on original questionnaire
Self-efficacy	57.1	79.8	0.864	0.418**[1]
Coping	38.8	93.0	0.658	0.604**[1] (P)
			0.618	0.700** (E)
			0.427	0.638**[1] (A)
Depression	40.0	100	0.840	0.775** [1]
Anxiety	47.4	97.2	0.558	0.653**[1]
Locus of control	55.4	64.2	0.087	0.342** (internal)
				-0.257** (physician)
				-0.080 (chance)
Social support[2]	100	34.4	0.724	0.652** (Oslo)
	N.A.	N.A.	0.629	0.591** (SSSS)
	N.A.	N.A.	0.742	0.723**[1] (total)
Perceived burden of disease[2]	N.A.	N.A.	N.A.	0.554**[1]

PPV: percentage of patients with a barrier on SeMaS that has a barrier according to the original questionnaire.
NPV: percentage of patients with no barrier on SeMaS that has no barrier according to the original questionnaire
Coping styles: (P) problem solving; (E) emotional; (D) distraction.
[1] Correlation: spearman's rho; sum scores were not normally distributed.
[2] The original questionnaires short scale of social support and EQ-5D do not use a division into categories.

Self-efficacy

The PPV for self-efficacy is 57.1%, and the NPV is 79.8%. To determine the cause of the low PPV, we analysed self-efficacy further. We computed cross tables on the two individual items of SeMaS with the two self-efficacy items from PAM-13, as these were very similar to the items from the perceived self-efficacy scale. The distribution is skewed, as most responses are in the positive response categories.

Some misclassification exists for the response category 'disagree' of the PAM questionnaire, versus 'somewhat true' of the SeMaS questionnaire, respectively 22 and 17 of the 204 responses for the two items. This results in suboptimal PPV and NPV values. Inter-item correlations between the SeMaS items and the PAM self-efficacy items ranged from 0.413 ($p<0.01$) to 0.461 ($p<0.01$) (data not shown).

As described in the methods section, the response categories for the self-efficacy questions were adjusted to improve internal consistency in the questionnaire. For the final instrument, we adjusted the response categories to the PAM instrument, giving less weight to the wish for internal consistency of the questionnaire. With this adjustment, we expect to improve the correlation, and decrease the misclassification.

Coping

The PPV of coping was 38.8%, and the NPV 93.0%. To further investigate the low PPV of the SeMaS coping subscale, we computed a cross table of the coping scales, which showed some misclassification of the coping styles. The scores on the SeMaS questionnaire showed 42 patients with multiple coping styles, while 18 patients had multiple coping styles on the UCL questionnaire. Also, SeMaS showed 29 patients with a distractive coping style, while the UCL showed 4 patients with this coping style.

To increase the PPV of the SeMaS, we added one item per coping style. We assessed the correlations between the UCL items and total scores per coping style. Correlations of the items for the problem solving coping style ranged from 0.621 to 0.860 ($p<0.01$). For emotional coping, the correlations ranged from 0.636 to 0.755 ($p<0.01$), and for distractive coping the correlations ranged from 0.761 to 0.819 ($p<0.01$). Per coping style, one item with a correlation >0.7 was added, based on face validity (problem solving: 0.813, $p<0.01$: emotional: 0.755, $p<0.01$; distraction; 0.761, $p<0.01$). The PPV changed from 38.8% to 41.5%, the NPV from 93.0% to 94.4%.

Depression

As shown in Table 1, the NPV of the depression subscale was 100%, and the PPV was 40.0%. When computing cross tables, we saw that 27 patients were categorised by SeMaS as having a minor or major barrier, while being categorised as having no barrier by the 4DSQ questionnaire, as shown in Table 2.

Table 2. Cross table of categories of depression

		4DSQ questionnaire depression			Total
		No barrier	Minor barrier	Major barrier	
SeMaS	No barrier	147	0	0	147
	Minor barrier	26	2	0	28
	Major barrier	1	8	8	17
Total		174	10	8	192

We assessed the effect of increasing the ‘no barrier’ category by one point. The misclassifications decreased by 15 patients, as shown in Table 3. Correlations between the SeMaS and 4DSQ categories increased from 0.667(p<0.01) to 0.785 (p<0.01).

Table 3. Cross table of categories of depression

		4DSQ questionnaire depression			Total
		No barrier	Minor barrier	Major barrier	
SeMaS	No barrier	164	1	0	165
	Minor barrier	9	1	0	10
	Major barrier	1	8	8	17
Total		174	10	8	192

We therefore increased the cut off point for the ‘no barrier’ category by one point. Furthermore, the practice nurses indicated in the interviews that patients experienced one of the items (‘last week, I had the thought “I wish I was dead”’) as shocking. We therefore assessed the correlations between the original items, and replaced this item in SeMaS by another item with a strong inter-item correlation (last week I thought life was not worth it; correlation 0.724, p<0.01). The NPV changed from 100% to 99.4%, the PPV from 40.0% to 67.9%.

Anxiety

The NPV of the anxiety subscale was 97.2%, and the PPV 47.4%. When computing cross tables, we saw the same pattern as in the depression domain: 34 patients were categorised as having a minor or major barrier by SeMaS, while being categorised as having no barrier by the 4DSQ questionnaire, as shown in Table 4.

Table 4. Cross table of categories of anxiety

		4DSQ questionnaire anxiety			Total
		No barrier	Minor barrier	Major barrier	
SeMaS	No barrier	144	0	0	144
	Minor barrier	32	2	1	35
	Major barrier	2	2	0	4
Total		178	4	1	183

We assessed the effect of increasing the cutoff point of the ‘no barrier’ category by one and two points. This decreased the misclassification of 4DSQ no barrier/SeMaS minor barrier from 32 to respectively 13 and 3 cases. The cross table of increasing this category by two points is shown in Table 5. Correlations between the SeMaS and 4DSQ categories increased

from 0.347 (p<0.01) to 0.580 (p<0.01). The NPV changed from 97.2% to 91.3%, the PPV from 47.4% to 77.8%.

Table 5. Cross table of categories of anxiety

		4DSQ questionnaire anxiety			Total
		No barrier	Minor barrier	Major barrier	
SeMaS	No barrier	173	0	1	174
	Minor barrier	3	2	0	5
	Major barrier	2	2	0	4
Total		178	4	1	183

Locus of control

For the dimension locus of control, the NPV was 64.2%, and the PPV 55.4%. These questions of SeMaS were analysed together with the data from the MHLCS. First, cross tables were made for the orientation scales of SeMaS and the MHLCS. The categories of SeMaS and MHLCS match partly, as shown in Table 6.

Table 6. Cross table of categories of locus of control.

		MHLCS				Total
		Internal orientation	Multiple orientations	Physician orientation	Chance orientation	
SeMaS	No barrier	49	6	11	26	92
	Minor barrier	22	8	19	17	66
	Major barrier	1	1	3	0	5
Total		72	15	33	43	163

Corresponding categories are: SeMaS ‘no barrier’ vs MHLCS ‘internal orientation’; SeMaS ‘minor barrier’vs MHLCS ‘multiple orientations’; SeMaS ‘major barrier’ vs MHLCS ‘physician orientation’ and ‘chance orientation’.

We used the available data to further investigate the correlations between the items and sum scores of the original MHLCS questionnaire. The SeMaS items correlate weak to moderate with the corresponding orientation. For the no barrier/internal orientation the correlation is 0.438 (p<0.01), for external/physician 0.349 (p<0.01), and external/chance is 0.067 (p>0.05). The original MHLCS items showed stronger correlations. For the internal orientation, the original MHLCS-item had a correlation of 0.817 (p<0.01) with the total score on this orientation. For the external/physician orientation, the original MHLCS-item had a correlation of 0.767 (p<0.01) with the total score on this orientation. Therefore, we adjusted the SeMaS

items to the original wording of the MHLCS items. The response categories were adjusted to the PAM. These categories are more consistent with the original MHLCS response categories, and this contributes to the internal consistency of the instrument.

Social support

For social support, two questionnaires were used. The Oslo 3-item social support scale consists of three questions about number of close confidants, sense of concern or interest from other people, and relationship to neighbours [1, 2]. The Short Scale of Social Support (SSSS) consists of 5 questions that measure actual support in case of need [3]. Both social support questionnaires were used in the SeMaS to be able to collect data and, if possible, choose one of the two for the measurement of social support. The wording of the Oslo 3-item social support scale was slightly altered, based on feedback from the patient focus group interviews. One item on the support from neighbours was added to the SSSS subscale, as this was relevant for the Dutch context.

The PPV and NPV for social support could only be computed for the Oslo 3-item social support scale, since the SSSS does not use a categorisation. The NPV for the Oslo-items was 34.4%, and the PPV was 100%. Inter-item correlations between two of the SeMaS-items and the original Oslo-items were low: 0.302 ($p<0.01$) and 0.285 ($p<0.01$), possibly caused by alteration of the wording. One item showed a strong correlation of 0.790 ($p<0.01$).

The inter-item correlations between the SeMaS items and original SSSS items are moderate to strong, ranging from 0.577 to 0.888 ($p<0.01$). In the SeMaS, the responses to the social support items show a higher number of missing values (n=140-200) than the other items. This is mainly the case for the items derived from SSSS.

If possible, we wanted to choose one of the social support measures for the SeMaS instrument based on the collected data, as described in the measures section. Since the inter-item correlations of the SeMaS and SSSS were better than the Oslo 3-item social support scale, we decided to incorporate the SSSS in SeMaS. To decrease the number of missing values, we adjusted the lay-out of the questionnaire.

PAM-13

First, using univariate ANOVA we investigated which subscales of the SeMaS should be included in the regression model. The subscales functional status, locus of control, self-efficacy, social support, anxiety and depression showed significant different PAM scores per SeMaS category and were therefore included in a multiple regression model. With the PAM scale as dependent variable, the forced entry regression showed that all above-mentioned subscales of SeMaS explained 21.9% (r^2) of the variance in the PAM score. Except for

perceived burden of disease, all subscales had a positive relation with the PAM scale. After the minor adjustments, the r^2 increased to 31.7%

Literature

1. Dalgard OS: **Community health profile as tool for psychiatric prevention**. In: *Promotion of mental health.* Edited by Trent DR, Reed CA, vol. 5: Avebury; 1996: 429.
2. Nosikov A, Gudex C, World Health Organization. Regional Office for Europe.: **EUROHIS : developing common instruments for health surveys**. Amsterdam ; Washington, D.C. Tokyo: Published on behalf of the World Health Organization. Regional Office for Europe by IOS Press ;Ohmsha; 2003.
3. Funch DP, Marshall JR, Gebhardt GP: **Assessment of a short scale to measure social support**. *Social science & medicine* 1986, **23**(3):337-344.
